# Hepatic Fat Accumulation Is Modulated by the Interaction between the rs738409 Variant in the *PNPLA3* Gene and the Dietary Omega6/Omega3 PUFA Intake

**DOI:** 10.1371/journal.pone.0037827

**Published:** 2012-05-21

**Authors:** Nicola Santoro, Mary Savoye, Grace Kim, Katie Marotto, Melissa M. Shaw, Bridget Pierpont, Sonia Caprio

**Affiliations:** Department of Pediatrics, Yale University School of Medicine, New Haven, Connecticut, United States of America; Scientific Directorate, Bambino Hospital, Italy

## Abstract

**Background:**

A single nucleotide polymorphism (SNP), the rs738409, in the patatin like phospholipase 3 gene (*PNPLA3*) has been recently associated with increased hepatic steatosis and ALT levels in adults and children. Given the potential role of *PNPLA3* in fatty liver development, we aimed to explore whether the influence of *PNPLA3* genotype on hepatic fat in obese youth might be modulated by dietary factors such as essential omega polyunsaturated fatty acids (PUFA) intake.

**Materials and Methods:**

We studied 127 children and adolescents (56 boys, 71 girls; 58 Caucasians; 30 African Americans and 39 Hispanics; mean age 14.7±3.3; mean BMI 30.7±7.2). The dietary composition was assessed by the Nutrition Data System for Research (NDS-R version 2011). The patients underwent a MRI study to assess the liver fat content (HFF%), ALT measurement and the genotyping of the rs738409 SNP by automatic sequencing.

**Results:**

As previously observed, HFF% and ALT levels varied according to the genotype in each ethnicity. ALT levels and HFF% were significantly influenced by the interaction between genotype and omega-6/omega-3 PUFA ratio (n-6/n-3), p = 0.003 and p = 0.002, respectively. HFF% and ALT levels were, in fact, related to the n-6/n-3 consumption only in subjects homozygote for the G allele of the rs738409 (r2 = 0.45, p =  0.001 and r2 = 0.40, p = 0.006, respectively).

**Conclusions:**

These findings suggest that the association of a high dietary n-6/n-3 PUFA with fatty liver and liver damage in obese youths may be driven by a predisposing genotype.

## Introduction

Non alcoholic fatty liver disease (NAFLD) is emerging as one of the most common complications of childhood obesity. It is associated with and predicts the metabolic syndrome, independent of overall obesity [1]. Recently, a non-synonymous SNP (rs738409), characterized by a C to G substitution encoding an isoleucine to methionine substitution at the amino acid position 148 in the patatin like phospholipase 3 gene (*PNPLA3*), was found to be associated with hepatic steatosis in a multiethnic cohort of adults [2] as well as in children [3], [4]. Moreover, it has been shown that this variant interacts with environmental stressors, such as obesity [5], [6] and alcohol consumption [7], that induce fatty liver. Indeed, these stressors seem to uncover the association between the rs738409 minor allele (G) and hepatic injury in populations in whom it is otherwise covert [8]. Interestingly, the same process occurs with some nutrients; the association between the *PNPLA3* variant, in fact, seems to be exacerbated by the total carbohydrate as well as total sugar intake [9].

Recently two studies have started pinpointing the physiologic role of *PNPLA3* and how this is affected by the rs738409 minor allele [10], [11]. In particular, it has been shown that the rs738409 minor allele is associated with a reduced hydrolytic capability of the protein [10] and that free fatty acids (FFA) potentiate the effect of *PNPLA3* rs738409 variant on intra-hepatic triglycerides accumulation [11]. This latter observation is of particular interest if we consider that the amount and the type of FFAs provided by the diet seem to play a pivotal role in the development of NAFLD [12]. Recent literature provides clues that the dietary imbalance between omega-6 (n-6) and omega-3 (n-3) polyunsaturated fatty acids (PUFAs) leads to development of an adverse cardiovascular and metabolic profile and contribute to the pathogenesis of NAFLD [13]. Biochemical analyses have shown alteration in the hepatic long chain fatty acid composition toward an increase in the n-6/n-3 PUFA ratio [14] and animal studies demonstrated that an excess of n-6 PUFA in the liver is associated with a pro-inflammatory state [15], [16] and an increased lipogenesis leading to massive fat accumulation into the liver [17]–[19]. Given these observations, in the present study we examined whether the association between the dietary composition in n-6/n-3 PUFA ratio and MRI measured hepatic fat accumulation might be influenced by the individual *PNPLA3* rs738409 genotype.

## Materials and Methods

### Subjects

We studied 127 children and adolescents (56 boys, 71 girls; 58 Caucasians; 30 African Americans and 39 Hispanics; mean age 14.7±3.3; mean BMI 30.72±7.23) from the New Haven area (New Haven, CT) recruited from the Yale Pediatric Obesity Clinic. Results from an oral glucose tolerance test performed as previously described [3] revealed that 97 (76%) had a normal glucose tolerance (NGT), 27 (21%) impaired glucose tolerance (IGT) and 3 (3%) type 2 diabetes (T2D). The study was approved by the Yale University Human Investigation Committee. Written parental informed consent and written child assent were obtained from all participants. All clinical investigations have been conducted according to the principles expressed in the Declaration of Helsinki.

### Genotyping and Liver Function

Genomic DNA was extracted from peripheral blood leukocytes. The *PNPLA3* rs738409 variant was genotyped by automatic sequencing as previously reported [3], [20]. Liver enzymes were measured using standard automated kinetic enzymatic assays also previously reported [3], [20].

### Dietary Composition

The dietary fat composition was collected and analyzed using the Nutrition Data System for Research (NDS-R versions 2006 and 2011), a software program developed by the Nutrition Coordinating Center (NCC), University of Minnesota. Subjects were instructed by a registered dietitian (RD) on completing a 3-day food record and advised to record two week days and one weekend day (since people generally eat differently on the weekends). No dietary instruction was given to the patients regarding dietary fat prior to the completion of the food records. The subjects returned their food records at MRI where dietitian checked the food records for accuracy, including specific quantity, product brand name, or cooking method details (NDS-R requires this multiple-pass method for data input). Final calculations were completed using NDSR version 2011. The ratio between omega 6 (n-6) and omega 3 (n-3) (n-6/n-3) PUFA was calculated by dividing the mean n-6 by the mean n-3 PUFAs.

### Abdominal Magnetic Resonance Imaging (MRI)

MRI studies were performed on a GE or Siemens Sonata 1.5 Tesla system [1], [3], [20]. Measurement of liver fat content was performed by MRI using the 2-point Dixon (2PD) method as modified by Fishbein et al [21]. Using the MRIcro software program, five regions of interest were drawn on each image and the mean pixel signal intensity level was recorded. The Hepatic Fat Fraction (HFF %) was calculated in duplicate from the mean pixel signal intensity data using the formula: [(Sin-Sout)/(2×Sin)]×100. The imaging parameters included: matrix size  = 128×256, flip angle (α)  = 30°, TR  = 18 ms, TEs  = 2.38/4.76 ms out-of-phase and in-phase, respectively, bandwidth  = 420 Hz/pixel, six averages, slice thickness  = 10 mm, one slice, 2.3 seconds/slice (for 2 points), scan time  = 14 seconds in a single breath-hold [22].

*Validation of Fast-MRI* against 1H-NMR was performed in 34 lean and obese adolescents. A strong correlation was obtained between the two methods (*r*  = 0.954, *p*<0.0001) [22]. The within-subject standard deviation for HFF was 1.9%. Recently, Pacifico et al reported that MRI measured liver fat content strongly correlates with macrovesicular steatosis/NASH (r = 0.86, p = <.009) in obese kids receiving a liver biopsy for diagnosis purposes [23].

### Statistical Analyses

The chi-square test was used to assess whether the genotypes were in Hardy Weinberg equilibrium and to test differences in genotype distribution as well as in gender and categories of glucose tolerance prevalence. Prior to analyze the data all the variables were tested for normality, with non-normally distributed variables log transformed to be better approximated by normality, except for HFF% for which a square root transformation was used. Differences in anthropometric features and in dietary intake among the groups of genotype were tested by ANOVA; age, gender, z-score BMI and ethnicity were used as covariates when appropriate.

Since the primary aim of the study was to explore whether the interaction between the rs738409 variant and n-6/n-3 PUFA could modulate hepatic fat accumulation in obese children and adolescents, the primary outcome of the study was the HFF%. In order to explore whether an eventual interaction may also drive to liver damage we tested as secondary outcome the ALT levels. ALT values were not available for 9 subjects (4 CC, 3 CG and 2 GG). To test the interaction between the genotype and dietary n-6/n-3 PUFA on the HFF% and ALT levels the subjects of the three ethnic groups were pooled, a regression coefficient (r^2^) was used and the genotype was coded with 0, 1, or 2 corresponding to the number of minor alleles carried by each individual. An interaction term between the rs738409 and the dietary intake of n-6/n-3 PUFA was added in a regression model in which age, gender, ethnicity, z-score BMI and glucose tolerance were used as covariates. Unless otherwise specified, for all the data raw means and standard deviations are shown.

## Results

The frequency of the minor allele for the rs738409 was 0.26 in Caucasians, 0.20 in African Americans and 0.49 in Hispanics (p = 0.01). The allele frequencies were consistent with those shown in similar ethnic groups in the Allele Frequency Database (ALFRED, http://alfred.med.yale.edu) as well as in HAPMAP (http://hapmap.ncbi.nlm.nih.gov/). Within each ethnic group there was no evidence against the null hypothesis that the genotype distribution was in Hardy Weinberg equilibrium for all of the variants (all p>0.05).

Table 1 shows the clinical features of the study population according to the genotype in each ethnic group. The three groups of genotype did not differ in terms of age, gender, z-score BMI and glucose tolerance in each ethnic group. As previously observed, HFF% varied among the *PNPLA3* rs738409 groups of genotype (Caucasians p = 0.0006, African Americans p = 0.001, Hispanics p = 0.09) [3], and there was a trend toward increased ALT levels in subjects homozygous for the G allele.

**Table 1:** Clinical features of the study population according to ethnicity and *PNPLA3* rs738409 genotype.

	CAUCASIANS			
	CC (34)	CG (18)	GG (6)	p-value
Age (years)	15.68±3.28	13.76±3.62	13.91±2.47	0.12
Gender (M/F) %	41/59	33/67	33/67	0.83
GT (NGT/IGT/T2D) %	85/12/3	83/17/0	83/17/0	0.91
Energy intake (Kcal) *	2417.3±622.8	2379.3±561.9	2162.4±601.2	0.78
n-3 PUFA (g) *	2.24±1.71	2.11±0.72	1.20±0.43	0.14
n-6 PUFA (g) *	16.81±8.64	15.07±6.77	11.52±3.63	0.59
n-6/n-3 PUFA*	9.86±5.92	7.64±3.21	9.64±90	0.17
BMI (Kg/m2)	29.77±7.47	29.10±5.47	35.28±6.57	0.15
HFF%**	4.13±7.23	6.58±9.65	27.40±13.50	0.0006
ALT (UI/L)*	21.14±14.15	36.73±48.25	32.80±17.48	0.33

	AFRICAN AMERICANS			
	CC (19)	CG (10)	GG (1)	p-value
Age (years)	16.57±3.38	16.18±3.13	12.00	0.41
Gender (M/F) %	37/63	40/60	0/100	0.73
GT (NGT/IGT/T2D) %	84/16/0	70/30/0	100/0/0	0.58
Energy intake (Kcal) *	2358.5±602.8	2233.4±333.9	2446.5	0.77
n-3 PUFA (g) *	1.69±0.85	1.90±1.33	1.58	0.57
n-6 PUFA (g) *	14.27±6.64	12.83±7.32	16.11	0.97
n-6/n-3 PUFA*	9.49±4.20	7.38±2.41	10.19	0.49
BMI (Kg/m2)	32.43±7.41	31.97±8.49	38.19	0.15
HFF%**	0.65±1.10	2.30±3.05	39.4	0.001
ALT (UI/L)*	15.11±6.39	13.50±8.05	52	0.34

	HISPANICS			
	CC (13)	CG (14)	GG (12)	p-value
Age (years)	12.97±3.08	13.27±1.99	14.09±3.34	0.33
Gender (M/F) %	54/46	73/27	50/50	0.40
GT (NGT/IGT/T2D) %	62/38/0	80/20/0	42/42/16	0.11
Energy intake (Kcal) *	2168.4±344.1	2561.3±673.3	2572.9±753.5	0.31
n-3 PUFA (g) *	1.60±0.73	1.81±0.72	1.62±0.94	0.73
n-6 PUFA (g) *	14.29±11.73	14.99±5.82	14.16±6.96	0.69
n-6/n-3 PUFA*	8.85±5.15	8.65±2.65	8.79±2.91	0.74
BMI (Kg/m2)	31.05±6.19	29.39±8.34	29.64±5.87	0.51
HFF%**	8.45±9.40	9.96±13.57	15.59±13.28	0.096
ALT (UI/L)*	25.36±15.88	29.78±21.75	47.18±30.46	0.010

* = log transformed and adjusted for age, gender, BMI, glucose tolerance. ** = Square root transformed and adjusted for age, gender, BMI, glucose tolerance. GT  =  glucose tolerance. NGT =  normal glucose tolerance, IGT =  impaired glucose tolerance, T2D  =  type 2 diabetes.

In the whole population the mean caloric intake was 2377.7±580.9 Kcal, the mean intake of n-3 PUFA was 1.8±1.1 grams and the mean intake of n-6 PUFA was 14.8±7.9 grams, the mean dietary n-6/n-3 PUFA was 8.9±4.2. There was no difference among the genotypes for the energy intake (CC 2351.31±537.0 Kcal, CG 2393.64±553.0 Kcal, GG 2444±693.8 Kcal, p = 0.82), n-6 PUFA intake (CC 15.5±8.7 grams, CG 14.3±6.5 grams, GG 13.4±7.9 grams, p = 0.51) and n- 3 PUFA intake (CC 1.9±1.3 grams, CG 1.9±0.8 grams, GG 1.4±0.7 grams, p =  0.26). The mean dietary n-6/n-3 PUFA was 9.56±5.26 in CC, 7.8±2.9 in CG and 9.1±2.3 in GG (p = 0.10).

**Figure 1:**
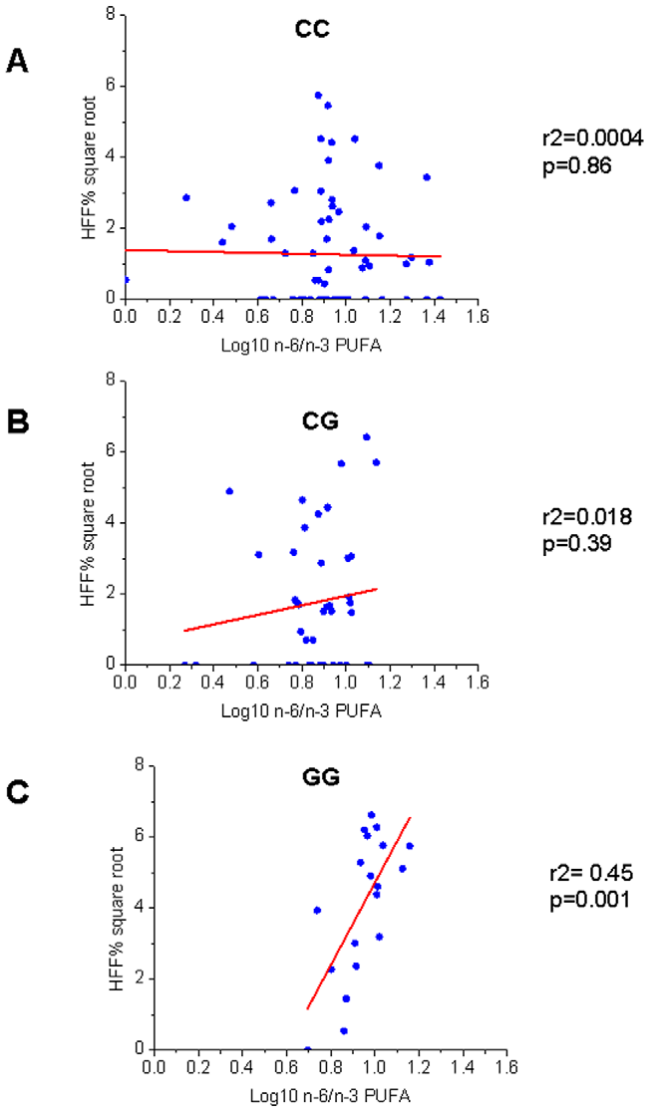
Interaction between *PNPLA3* rs738409 and n-6/n-3 PUFA in modulating HFF%. The figure shows a different degree of regression between HFF% (square root) and n-6/n-3 PUFA (log10) in the three genotypes. In the CC (Panel A) and CG (Panel B) group there was no association between HFF% and n-6/n-3 PUFA (r2 = 0.0004, p = 0.86 and r2 = 0.018, p = 0.39, respectively). Only in the GG group (Panel C) there was a strong association between HFF% and n-6/n-3 PUFA (r2 =  0.45, p = 0.001).

**Figure 2:**
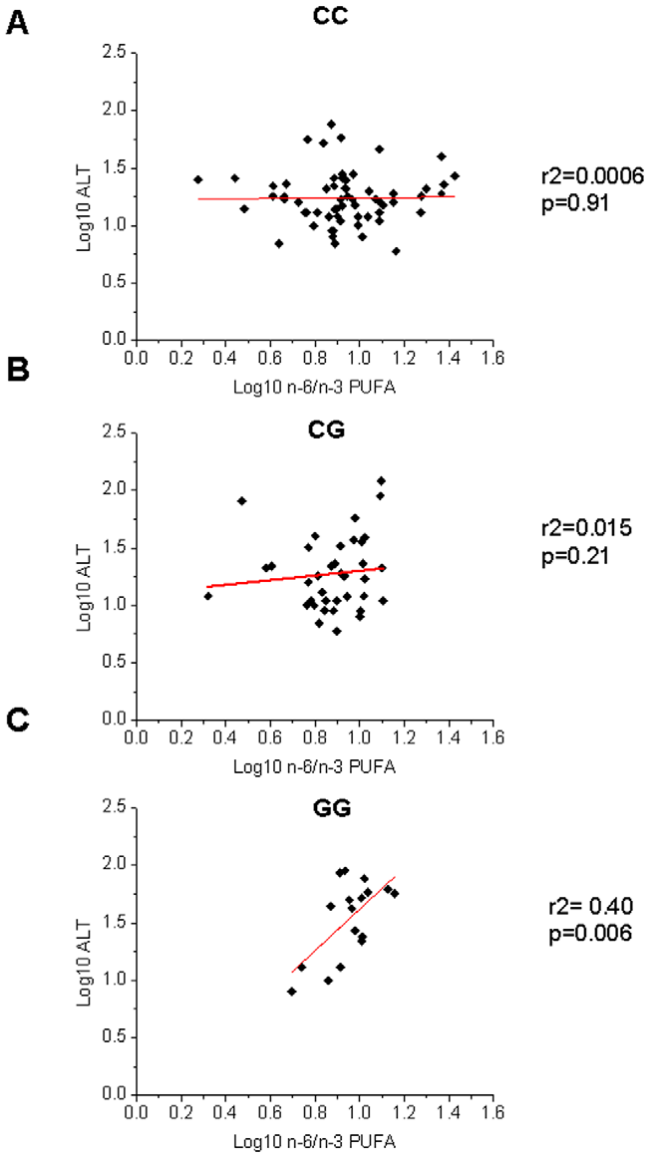
Interaction between *PNPLA3* rs738409 and n-6/n-3 PUFA in modulating ALT levels. The figure shows a different degree of regression between ALT (log10) and n-6/n-3 PUFA (log10) in the three genotypes. In the CC (Panel A) and CG (Panel B) group there was no association between ALT and n-6/n-3 PUFA (r2 = 0.0006, p = 0.91 and r2 = 0.015, p = 0.21 respectively). Only in the GG group (Panel C) there was a strong association between HFF% and n-6/n-3 PUFA (r2 =  0.40, p = 0.006).

We observed an interaction between the *PNPLA3* gene variant and the n-6/n-3 PUFA in influencing HFF% (p = 0.002) and ALT levels (p = 0.003), independently of age, gender, z-score BMI, ethnicity and glucose tolerance (p = 0.017 and p = 0.016 respectively). This interaction was the result of a different regression degree between the n-6/n-3 PUFA and HFF% or ALT in the groups of genotype. In fact, there was a strong association in the GG group between n-6/n-3 PUFA and ALT or HFF% (r2 = 0.40, p = 0.006 and r2 = 0.45, p = 0.001 respectively), while the same association was not present in the other two groups of genotype (CC and CG) (figure 1 and figure 2).

## Discussion

The main result of this study is the observation of an interaction between the *PNPLA3* rs738409 and n-6/n-3 PUFA on hepatic fat content and ALT levels. This novel finding helps to explain the strong association between the rs738409 SNP and NAFLD that has been largely and repeatedly demonstrated [2]–[8] and raises new questions about the function of the *PNPLA3* itself as well as about its potential implications in the development of cardiovascular diseases.

Since the association between the *PNPLA3* rs738409 and NAFLD has been discovered [2], several studies have tried to unravel the pathogenetic mechanism underlying this association. It has been suggested that this variant may cause a gain of function of the protein, which would act as a lipogenic factor [24]. In fact, while knock out mice for the *pnpla3* do not show any increased fat accumulation into the liver with respect to the wild types [25], [26], the administration through viral vectors of the mutated *PNPLA3* confers to wild type mice a higher susceptibility to fatty liver [24]. Consistently, it was also shown that *SREBP-1c*, activated by carbohydrate feeding, transcriptionally activates *pnpla3* as well as several genes encoding enzymes in the fatty acid biosynthetic pathway [23]. This mechanism seems also to be indirectly supported by studies showing an interaction between the carbohydrates intake and the *PNPLA3* rs738409 in determining the development of fatty liver [9]. The only data that do not fit with this hypothesis is the lack of association of the *PNPLA3* variant with increased plasma triglycerides [2], [3].

Our group has previously proposed another mechanistic hypothesis based on the observation that subjects carrying the G allele show smaller subcutaneous adipose cells. Since the *PNPLA3* has been suggested to be a potential growth factor for adipose cells [3], we suggested that in these subjects there might be an overflow of free fatty acids from the adipose tissue to the liver given the lower capacity of their adipose cells to store the FFA [3]. This hypothesis was based on a small sample of obese adolescents; studies in animals knockout for the *pnpla3* gene showed that the *pnpla3* does not contribute significantly to adipose cells development [25].

Perhaps more promising are the results provided by studies focusing on the hydrolytic action of the PNPLA3 given that the PNPLA3 along with the acylglycerol transacetylase activity also has a triacylglycerol hydrolase function [10]. By studying this latter mechanism, it has been recently suggested that the rs738409 variant might cause a lack of the PNPLA3 hydrolytic function [10]. In particular, the gene variant seems to lower the protein ability in hydrolyzing the n- 9 of about 15% [10]. The n-9 represents the most common fatty acids in the diet, deriving from meat, olive oil, sesame oil, almonds, and avocados, but they are also synthesized starting from essential polyunsaturated fatty acids such as the n-6 [27]. More recently, another study by Perttila et al has shown that the presence of a methionine in postion 148 in the PNPLA3 enhances the cellular accumulation of triglycerides in presence of an excess FFA by significantly slowing down the triglycerides hydrolysis [11]. Our observation seems to be consistent with these animal and in vitro studies [10], [11], [28] supporting indirectly the role of PNPLA3 in lipid hydrolysis. In fact, given those evidences, one could speculate that the overload of n-6 in the diet will serve both as substrate for new triglycerides and in the meantime will be slowing down or delaying the hydrolytic function of the PNPLA3. Thus, in subjects carrying the rs738409 minor allele, while the newly formed triglycerides will tend to accumulate into the liver leading to hepatic steatosis, the excess of n-6 not incorporated into triglycerides will lead to the over-synthesis of proinflammatory n-6 derived species, which in turn trigger the second hit responsible for the inflammation that leads to NASH.

Our observation of an interaction between the *PNPLA3* rs738409 variant and the high n-6/n-3 PUFA suggests that we could provide a targeted therapy to subjects with NAFLD homozygous for the minor allele either reducing the dietary n-6 amount or alternatively increasing dietary intake of foods rich in n-3 PUFA, such as salmon, tuna, and flaxseed oil, or supplementing the diet with n-3 PUFA. The omega 3 supplementation, in fact, by balancing the n-6/n-3 PUFA has been shown to be effective in reversing hepatic steatosis in animal as well as in humans [14], [29].

In conclusion, our findings show an interaction between the *PNPLA3* rs738409 variant and the dietary n-6/n-3 PUFA in modulating the hepatic fat accumulation and the liver damage in obese youths. The study findings generate new questions about the function of the PNPLA3 itself and pose new opportunity for targeted therapy in patients with NAFLD.
